# CBCT imaging: a simple approach for optimising and evaluating concomitant imaging doses, based on patient-specific attenuation, during radiotherapy pelvis treatment

**DOI:** 10.1259/bjr.20210068

**Published:** 2021-07-19

**Authors:** Caroline Ordóñez-Sanz, Mark Cowen, Neda Shiravand, Niall D MacDougall

**Affiliations:** 1 Radiotherapy Physics, St Bartholomew’s Hospital, London, UK; 2 North West Anglia NHS Foundation Trust, England, UK; 3 Barts Cancer Institute, Queen Mary University of London, London, UK

## Abstract

**Objectives:**

A simple, robust method, for optimising cone-beam CT (CBCT) dose and image quality for pelvis treatment, based on patient-specific attenuation.

**Methods:**

Methods were investigated for grouping patients into four imaging categories (small [S], medium [M], large [L], extra large [XL]), based on planning-CT CTDIvol, and phantoms constructed to represent each group. CBCTs with varying kV, mA and ms honed in on the best settings, with a bladder noise of 25 HU. A patient pilot study clinically verified the new imaging settings.

**Results:**

The planning CTDIvol is a reliable method for grouping patients. Phantom measurements from the S, M and L groups show doses significantly reduced (19–83% reduction), whilst the XL group required an increase of 39%. Phantom TLD measurements showed the number of scans needed to increase rectal organ at risk (OAR) dose by 1 Gy was 143 (S group) and 50 (M group). Images were qualitatively assessed as sufficient by clinicians.

**Conclusion:**

Patient-specific CBCT modes are in use clinically with dose reductions across all modes except Pelvis XL, keeping doses ALARP and images optimal. Consideration of OAR doses controls the number of CBCTs allowed to ensure adherence to OAR tolerance. Reporting CBCT doses in “scans per Gray” allows clinicians to make informed decisions regarding the imaging schedule and concomitant doses.

**Advances in knowledge:**

Patient grouping at planning CT, using CTDIvol, allows for CBCT imaging protocols to be selected based on patient specific attenuation. Reporting OAR doses in terms of “scans per Gray” allows translation of imaging dose risk to the Oncologist.

## Introduction

Radiotherapy treatments typically deliver between 2 and 6 Gy per day to treat patients with cancer. When dealing with such high radiation doses, safety and accuracy are paramount to ensure good patient outcomes. Over the last two decades, simple conformal treatments have been steadily discarded in favour of the more sculpted dose distributions available from intensity modulated radiotherapy (IMRT) and subsequently volumetric modulated radiotherapy (VMAT). The challenge in radiotherapy quickly changed from a planning challenge of how to plan dose coverage with conformal fields, to how to accurately deliver complex VMAT distributions. Encouraged by the availability of dose sculpting with IMRT and VMAT, the race was now on to improve the accuracy of the placement of these distributions within the patient. The only way to gain the millimetre accuracy required is to image the patient when they are set up in the treatment position. X-rays are the most widely available method for imaging and therefore come to the forefront in image-guided radiotherapy (IGRT).

The technology available to allow image-guided set up in radiotherapy has changed dramatically over this period. Where once mega voltage (MV) planar imaging was standard, the advent of kV imaging add-ons to linacs has allowed more useful (better contrast); lower dose and frequent imaging to be implemented. IGRT techniques means that patient-specific treatment can be adjusted due to observed anatomy changes. CBCT imaging is an extremely useful dosimetry tool which allows for monitoring of these changes and has opened up the potential for soft tissue matching, plan adaptation and monitoring of patient weight-loss. Thereby, increasing the possibilities for reduced set up margins^1^ and smaller treatment volumes, with the aim of reducing morbidity and increasing cure rates. Various work has demonstrated that it is possible, particularly in the case of pelvis radiotherapy (RT) treatments that daily CBCT imaging can lead to organ doses greater than 1 Gy if imaged every fraction.^2–13^ This includes Monte Carlo methods, dose calculators in addition to thermoluminescent measurements within anthropomorphic phantoms. Modelled and measured CBCT doses (using the manufacturer’s standard imaging protocols) stated in these publications should give cause for concern and highlights the increased awareness needed into CBCT concomitant doses within each hospital centre.^14^ Hess et al^15^ discuss promoting a culture and practice of *“gentle IGRT” ‘* a terminology which merits due consideration and every effort should be made to ensure that doses are kept ALARP.

## The need for optimisation

The need for imaging in radiotherapy has arguably driven ahead of the requirement for image optimisation. Barts Health NHS Trust, London, is a Varian centre with 3 TrueBeams and 2 Clinac iX (as of May 2020). Now that treatment imaging has been achieved, the focus is on optimising imaging dose and image quality. Linac CBCT systems do not automatically optimise kV dose, so radiotherapy departments are moving towards having one or more customised CBCT mode(s), optimised per anatomical site, with each mode having tailored kV and mA to account for different sizes of patient separation and anatomical variations.

At Barts Health NHS Trust, the CBCT imaging modes initially implemented (High-quality head, Low dose thorax, Pelvis Spotlight, Barts H & N) had been done so using the standard Varian OBI settings (with the exception of Barts H & N). Under IR(ME)R, doses must be kept ALARP and image quality must be sufficient for purpose. The dose was measured in each clinical mode and deemed clinically acceptable at commissioning. The images were also verified that they were of sufficient quality for clinical use. However, based on reports from other Varian centres,^16,17^ there is much scope for optimising the standard *“one size fits all”* manufacturer settings, both in terms of minimising patient dose whilst maintaining clinically acceptable images. In terms of optimisation, optimal CBCT images are those that satisfy the clinical need — not necessarily those which are of equal or better quality than using the standard Varian settings. Similarly, the aim of optimisation is not always to minimise the CBCT dose. For patients with a larger separation, the optimal dose settings are likely to be higher than the standard Varian settings. For pelvis treatment in particular, there is a strong correlation between BMI and organ doses^4,8^ and the suitability of one standard imaging setting for all, given the increased frequency, comes into question when keeping doses ALARP.

## Balancing risk and benefit of CBCT imaging

The measurements of CT dose index (CTDI) and cone beam dose index (CBDI) are useful for comparing different CBCT modes and aid in optimisation. However, they provide little information about the CBCT dose distribution within the patient. The risk *vs* benefits of imaging dose must be taken into account in the context of radiotherapy, this is quite different to diagnostic imaging. One important distinction is that the imaging dose associated with radiotherapy treatment is to enable accurate delivery of a much larger dose of radiation, so could be thought of as more akin to interventional radiology than diagnostic. Currently in the UK, there are no national diagnostic reference levels for CBCT exposures, though a national survey was conducted recently, so this may change. Until then, this places a greater need for departments to establish their own local reference levels in addition to performing audits of these doses with other centres. Whilst not a requirement under IR(ME)R, as it is for diagnostic imaging,^18^ this is regarded as good practice.^19^ More recently, there had been an update to IR(ME)R 2017 regulations to include reporting criteria for radiotherapy treatment verification imaging. Significant accidental or unintended exposures (SAUE) need to be notified to the enforcement authority under Regulation 8 (4) which has led to limits now placed on the number of repeat CBCT images that can be acquired during the course of a patient’s treatment.

At Barts Health NHS Trust, CBCT imaging is being used to improve patient setup and treatment accuracy. CBCT is so useful that it is quickly becoming needed more than once per fraction for difficult patient setups. During the CBCT imaging procedure, large portions of radiosensitive structures are irradiated, this additional imaging dose adds to the therapeutic radiation dose. Assuming all CBCTs taken are clinically justified, is there a number of CBCTs that are too many? Is it possible to exceed sensitive organ tolerance? Hence as more imaging schedules move toward daily CBCT, it is important that the doses to organs at risk (OARs) are evaluated too.

One cannot assume that published doses are what your Linac will be producing. Indeed, in the process of optimising our CBCT scans we have developed custom modes that are lower in dose and comparable in image quality to the default Varian modes. In the optimised CBCT modes for each specific IGRT process, the associated dose will vary with Linac type, software version, imaging geometry, filtration, exposure parameters, reconstruction algorithms, number of scan projections.^7,8,20,21^

This work sets out a simple method for implementing pelvis CBCT imaging protocols, based on patient size, for the reduction in concomitant imaging doses. With the patient demographic varying between hospital centres, it makes sense to adapt the CBCT imaging settings by representatively grouping the patients based on radiation attenuation. That way the images can be optimised more specifically to the most common groups of patients within a department. This work also addresses the importance of performing departmental dose (OAR and CTDIw100) measurements for each imaging CBCT mode. Discussions are given on how we can translate these results into a more meaningful method for making judgements, and of course justification, of additional imaging in the context of individual patient plans. All work discussed here, can be easily implemented within a centre using equipment common to radiotherapy departments.

## Methods and materials

**Figure 1.**
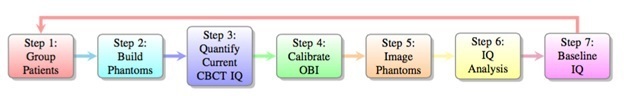
Steps implemented for optimising CBCT imaging protocols. CBCT, cone beam CT.

Figure 1 gives an overview of the steps necessary for implementation of the dose optimising method for the pelvis CBCT imaging protocols at Barts Health NHS Trust.

- Group patients into four build categories.
- Build phantoms representative of each group.
- Quantify the current CBCT image quality for use as a comparative baseline.
- Calibrate the OBI for several kV beam spectra.
- Image S/M/L/XL phantoms under different kV beam conditions.
- Perform image quality analysis and determine optimum imaging settings.
- Establish base line image quality parameters for each imaging protocol.

### Grouping patients based on build

An audit was carried out on 110 pelvis patients in order to find the best method for grouping patients based on build. Bart’s used a GE-16 slice CT scanner and for each patient, the planning CT scan images were analysed and the widest CT slice noted, with corresponding mA. The summary dose report, output by the scanner, for each patient, was also viewed and the corresponding CTDIvol (mGy) recorded. It’s useful to note that this value uses the average mA from the entire scan to determine a whole body dose value.

Next, four imaging phantoms (S, M, L, XL), representative of the upper limits of CTDIvol in each group were constructed. This was achieved using the Catphan^®^, Barts solid water and vaseline bolus together with a bespoke annulus to increase separation, building on patient size. Various combinations were made and then scanned in order to confirm that the CTDIvol was consistent and appropriate for each imaging group

### Quantify current CBCT image quality

The 110 pelvis patients were analysed further in order to quantify the current CBCT image quality obtained from the Varian “Pelvis Spotlight” images. For this purpose, a region of interest within the patient’s bladder was analysed and the noise level (Stdev HU) noted. For comparison, the GE-16 slice planning CT scanner uses an inherent 16 HU noise value, whereby the scanner changes the mA at each imaging slice in order to maintain a constant noise level at the imaging panel.

### Calibrate OBI imaging panels

Varian Spotlight imaging used a 125 kV beam spectrum. However, to further optimise the dose and image quality a range of beam spectra is necessary so that the most suitable beam quality can be selected based on patient attenuation. The kV of the beam spectra selected in this work for calibration of pelvis modes were 125, 110 and 100 kV. The Varian OBI imaging calibration protocol was carried out for each kV and included: Dual Gain calibration, I0 calibration, Normalisation and HU calibration. This procedure was repeated across all Varian treatment machines. In addition, the new pelvis imaging modes were edited to provide a larger field of view. For TrueBeam linacs, the Varian software does not allow for commissioning a 110 kV beam so the 125 kV beam was optimised for this patient groups L, XL and a 100 kV beam for the S and M categories.

### Imaging optimisation

Four patient-size phantoms were imaged separately and the exposure settings (mA, mS) for each kV were varied. The Catphan^®^ uniformity section was analysed within each phantom and the exposure settings that gave a reference CBCT noise level of 25 HU within a consistent ROI (comparable to the bladder noise) was selected as optimal. In addition, baseline image quality results were recorded, as per monthly imaging QA Catphan^®^ tests, for each kV, across all treatment linacs, in order to assess that image quality was consistent and within tolerance. This included spatial resolution, low contrast detectability, in-plane spacial integrity, HU accuracy and image uniformty. The Diagnostic Radiology department within Barts Health NHS Trust performed ‘CTDIw100 type’ measurements using central and peripheral dose measurements in a 32 cm PMMA body phantom and 100 mm ionisation chamber.

### Clinical pilot study

Having carried out our imaging optimisation for each group using patient-sized phantoms, the next step was to carry out a clinical pilot study. A number of patients were selected to be scanned using the standard Spotlight CBCT images in addition to the new protocol imaging settings during their first few treatment fractions. These were then visually assessed by both clinicians and radiographers in addition to quantitative analysis of the bladder noise for comparative purposes of image quality. To further quantify the effect of this work, a retrospective audit on 110 patients was carried out to ascertain the level of dose reduction to the Barts’ patient demographic.

## Results

### Grouping patients based on build

**Figure 2.**
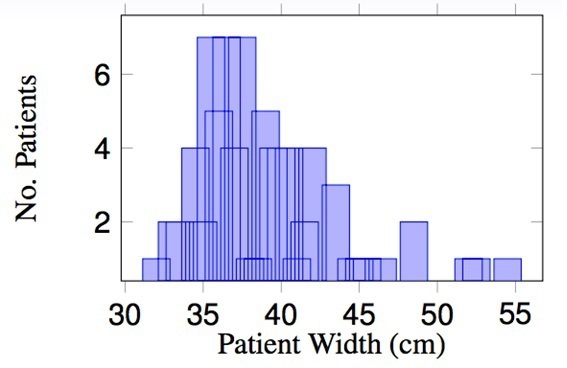
Patient width (cm) at the widest CT slice.

**Figure 3.**
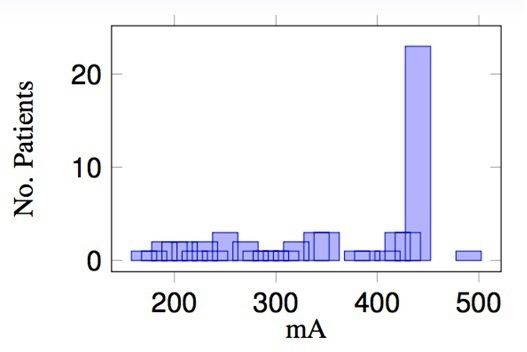
mA associated with the widest CT slice for each patient.

**Figure 4.**
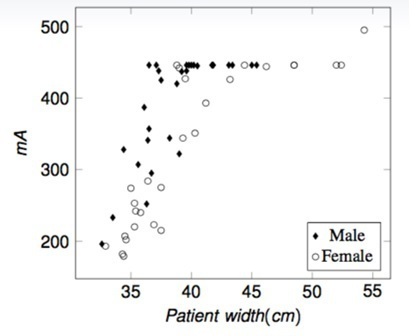
mA (widest slice) versus patient width (cm).

Figure 2 shows the patient data binned on the widest CT slice manually assessed by scrolling through the planning CT scan data set. From this plot, there is no correlation to allow groups to be selected: the widest slice is not representative of the patient’s overall body volume. Figure 3 shows the patient data plotted for the mA attained at the widest scan slice. There is a peak at 446 mA which is the value at which the scanner tube current saturates unless a manual override is performed. Similarly, no obvious groups could be easily extracted from these data. The data were further examined and a plot of mA *vs* patient thickness, noting gender, carried out (Figure 4). The results highlight the different anatomy and body density compositions between both male and females and the significance in not using mA or patient thickness as a grouping indicator.

**Figure 5.**
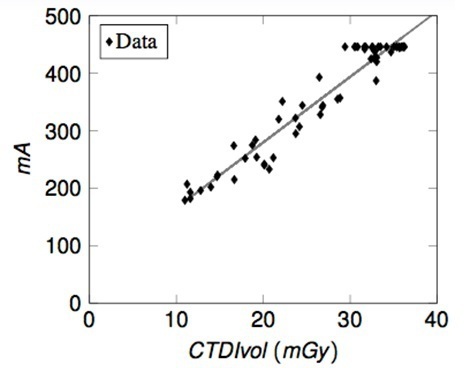
CTDIvol *vs* mA (widest slice). CTDI, CT dose index.

Next, the CTDIvol dose indicator was plotted against the widest slice mA. It shows a good, linear correlation between the two parameters, making it a consistent and reliable method for grouping patients based on build. Figure 5 shows the results. Four different groups were identified and are summarised in Table 1. During pre-treatment checks, the radiographers identify the patient’s CBCT imaging group. It should be noted that any upgrade to the planning CT scanner, will require issuing of new CTDIvol values. At Barts Health NHS, new values were issued in February 2020 following the installation of a new Siemens CT scanner where the newer technology gave rise to lower CTDIvol values.

**Table 1.** CTDIvol patient grouping

Group	CTDIvol (mGy)
Pelvis S	<15
Pelvis M	15.1–24
Pelvis L	24.1–36
Pelvis XL	>36.1

CTDI, CT dose index.

### Quantify current CBCT image quality

**Figure 6.**
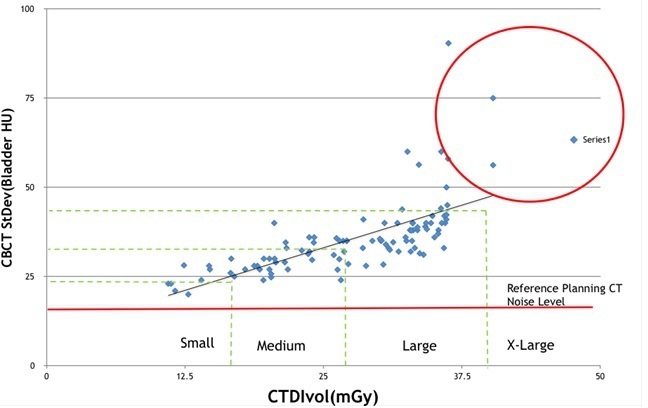
CTDIvol *vs* image noise within bladder.

A plot of the CTDIvol (mGy) *vs* bladder noise shows that as the patient’s size increases, so too, does the imaging noise, as we would expect (Figure 6). The reference planning noise level is given as the solid line at 16 HU for comparison. For patients who were classified as X-Large, the noise values increase above 50 HU and in consultation with the radiographers, these patients were most challenging when performing an online bony match using the Varian software.

### Calibrate OBI panel

**Figure 7.**
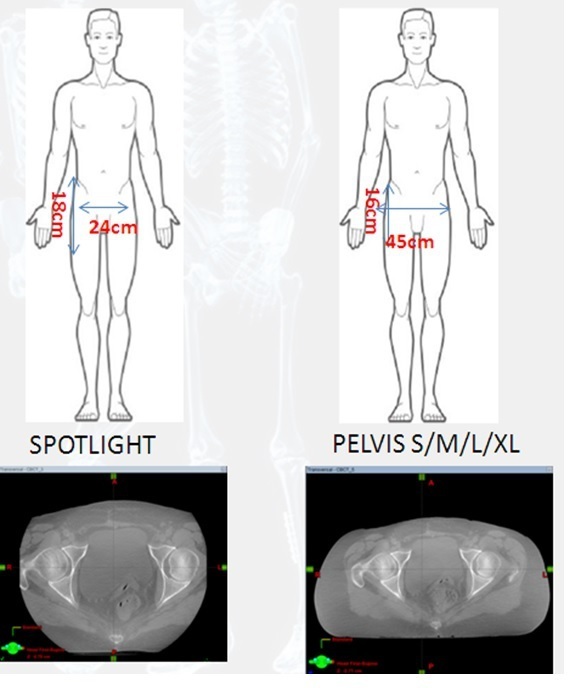
Summary visual of FOV changes for the optimised pelvis imaging modes. FOV, field of view.

New pelvis imaging modes were created to provide a larger field of view to allow for more OARs to be visualised as well as the external patient contour (45 × 16 cm (New protocol) *vs* 24 × 18 cm (Pelvis Spotlight)). Figure 7 provides a visual of these changes to imaging protocol. A full body contour scan is acquired using 360 degrees and a half fan acquisition. This is in comparison to the Varian Spotlight mode that consisted of a 200 degree scan rotation and full fan acquisition. These changes allow for more useful body contour checks throughout the patient’s treatment and for easier decisions on replanning scans for those having lost too much weight.

### Imaging optimisation

Whilst these measurements were by no means an accurate representation of the true dose within the phantom due to various factors^14^ (scatter conditions not exact, beam length longer than phantom, only measures an average of the central portion of the central axis), they did provide a comparative aid in the assessment of the total scan dose for each imaging protocol. Table 2 summarises the optimal exposure settings for each patient group and their respective doses when compared to Varian Spotlight settings, as measured on the Clinac iX. The doses shown in Table 2 are whole body doses and are expressed in terms of dose per imaging scan and as a total dose over a prostate and nodes 37 fraction pelvis treatment. TheTrueBeam imaging protocol for Pelvis L used 125kV and reduced exposure settings of 80 mA and 16 mS as a compromise to the 110 kV beam settings used on iX Clinacs. The standard Varian Pelvis Spotlight settings give a 1.1 Gy concomitant dose over a 37 fraction pelvis treatment. Pelvis small, medium and large imaging doses give −83.2%, −56.5%, −19.2% less dose respectively. Pelvis XL gives a 39.4% increase in dose when compared to Varian’s, however imaging noise was improved and optimised (25 HU) for online matching.

**Table:** Table 2: Summary of imaging settings and whole body doses over a typical 37 fraction treatment course measured on the Clinac iX machines. TrueBeam imaging dose parameters are included, however CTDI phantom measurements were not obtained. Instead air kerma measurements were taken for each imaging protocol on both Clinac and TrueBeam machines and a further dose reduction of 50% for TrueBeam is inferred from these results (see Table 3).

Group	kV	mA	mS	mAs	CTDIw100	Dose (cGy)	Dose diff (%)	Dose (37#) (Gy)
					(mGy/mAs)			
Clinac iX Imaging Parameters								
Spotlight	125	80	25	704	4.09	2.9	0	1.1
Pelvis S	100	40	12	609	1.59	0.5	−83.2	0.2
Pelvis M	100	63	20	1014	1.57	1.3	−56.5	0.5
Pelvis L	110	80	20	1013	2.3	2.3	−19.2	0.9
Pelvis XL	125	80	25	1266	3.17	4	39.4	1.5
TrueBeam Imaging Parameters								
Pelvis S	100	40	12	317				
Pelvis M	100	63	20	832				
Pelvis L	125	80	16	845				
Pelvis XL	125	80	25	1320				

CTDI, CT dose index.

**Table:** Table 3: Comparison of isocentre air kerma measurements for each clinical mode using a 100mm ionisation chamber on both the Clinac iX and TrueBeam linacs.

Imaging Protocol	Air Kerma (mGy)		Air Kerma Ratio (TrueBeam/Clinac iX)
	Clinac iX	TrueBeam	
Pelvis S	22	10.8	49%
Pelvis M	57.9	27.5	47%
Pelvis L	89.3	50.1	56%
Pelvis XL	146.6	77.5	53%

To quantify the difference in dose for each imaging protocol between the TrueBeam and Clinac iX, air kerma measurements were taken using a 100 mm ionisation chamber placed at the imaging centre. Table 3 summarises these results and it is shown that the doses measured for TrueBeam are approximately 50% lower than the Clinac iX machines, consistent across all imaging modes. This is due to advancement in technology with the increased efficiency of the detector imaging panel, a harder kV beam provided by the titanium filter, in addition to the advanced reconstruction algorithms applied to the acquired images.^22^ This is another important factor that can be considered when justifying additional images.

### Clinical pilot study

**Figure 8.**
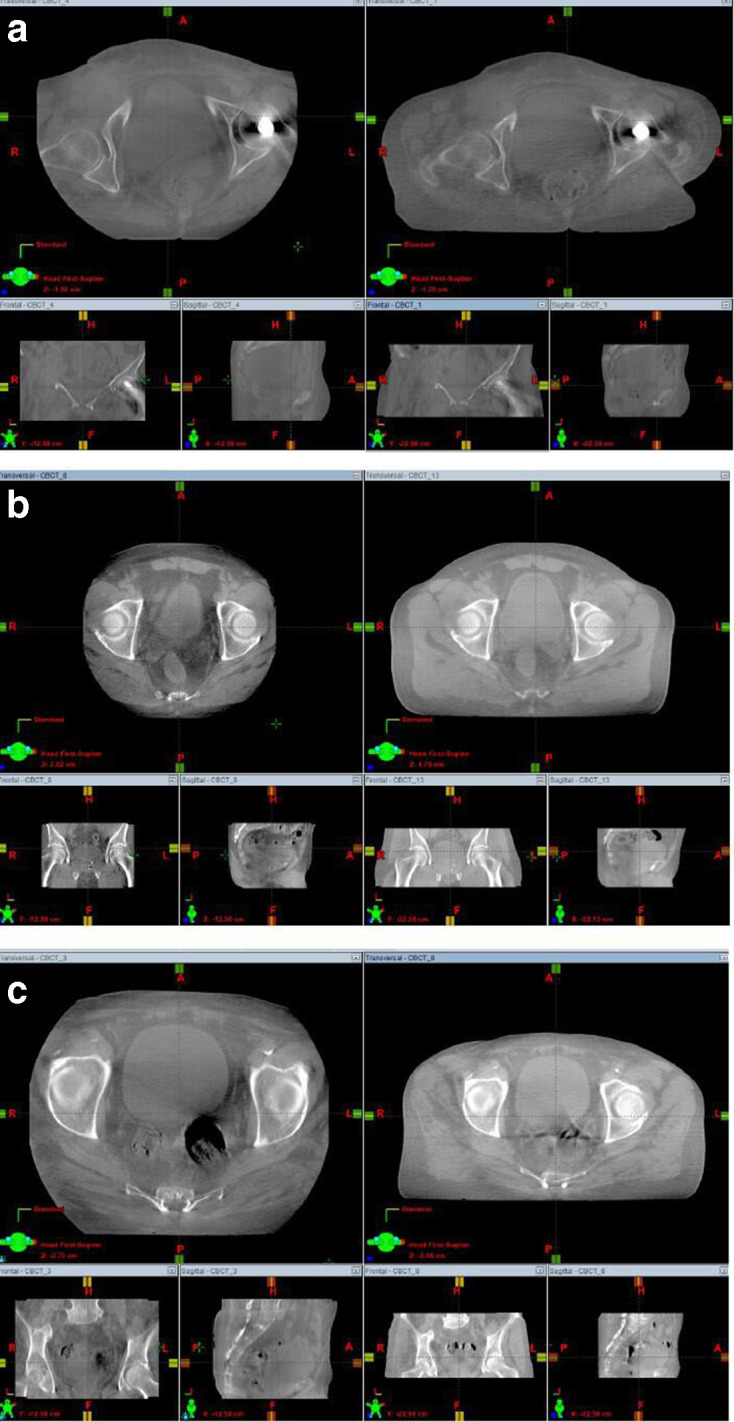
(a) Comparison of a small category patient. Varian Spotlight (left) *vs* optimised pelvis S settings (right). 83% dose reduction achieved. (b) Comparison of a medium category patient. Varian Spotlight (left) *vs* optimised Pelvis M settings (right). 57% dose reduction achieved. (c) Comparison of a large category patient. Varian Spotlight (left) *vs* optimised Pelvis L settings (right). 19% dose reduction achieved.

During the clinical pilot study, patient images were acquired for patient groups, S, M and L on the Clinac iX linacs. No XL build patients were recruited during the clinical pilot. Patient images within the Pelvis S category can be seen in Figure 8a, with the standard Spotlight settings on the left and the optimised images on the right. Clinicians deemed the optimised images acceptable in image quality and adequate for purpose. Noise levels for both the Spotlight and Pelvis S images were comparable, both achieving a noise level range within the bladder of 25–30 HU. However, the dose savings were vast with the newly optimised images acquired at 83% less dose. Figure 8b shows an image comparison for Spotlight versus the new optimised Pelvis M settings. Again, these images were deemed of sufficient quality, however the optimised images use 57% less dose than the standard Spotlight settings. Noise levels were also similar for both images at 25–30 HU. Lastly, Figure 8c shows the results for a patient within the large category. The noise levels for Spotlight were lower (15 HU) compared to the newly optimised settings (30 HU). However both images were deemed clinically acceptable with the optimised images giving 19% less dose than the Varian standard.

**Figure 9.**
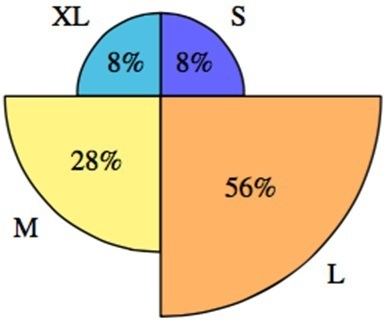
Retrospective audit showing the categories that patients would have been imaged in. 92% of patients would have had some form of dose reduction to their CBCT imaging regime.

The retrospective audit of 110 patients shows the level of dose reduction to the Barts’ patient demographic. Figure 9 demonstrates that 92% of patients would have benefitted from a reduction in dose (varying from 19 to 83%) whilst 8% required more dose (39%) to achieve images of sufficient quality.

## Discussion

St Bartholomew’s Radiotherapy department has implemented and used the new pelvis CBCT imaging settings clinically for the last 6 years. This work has aided the department’s confidence in carrying out daily CBCTs for the majority of pelvis patients, in comparison to just once weekly, prior to this work being started. The use of routine CBCT imaging has proven immensely helpful in patient pre-treatment setup, in evaluating patient weight-loss and the need for replanning a patient’s treatment. Further work for CBCT protocol optimisation has also been developed to other anatomical sites, such as head and neck treatments.^23^

The benefits of on-treatment imaging are visual, the risks less so. Also, how does one translate this imaging dose risk to the Oncologist so they can justify the imaging dose? The last stage of our work attempts to address this problem by using a simple data format methodology that allows clinicians to make informed decisions regarding the imaging schedule and justification of concomitant doses. It involves the consideration of the rectal OAR dose within pelvis RT treatments. Over the last 6 years, we have developed a streamlined methodology for measuring OARs for clinical CBCT modes, using equipment readily available in most RT departments. Further work by Shiravand et al^24^ at Barts Health measures the absorbed doses from CBCT to OARs using an anthropomorphic phantom (ATOM^®^) and TLDs, placed at the position of the rectum and the phantom irradiated with the selected Pelvis CBCT mode. Doses were measured using TLDs, for Pelvis Small and Medium clinical CBCT modes. Note that the ATOM^®^ phantom is the size of a small person, so measurements will overestimate the dose to a medium-sized person. The average OAR dose per scan for the pelvis ranged from 0.17 to 1.96 cGy. Table 4 reports the results for a prostate plus nodal treatment plan, in terms of the number of CBCT scans that would deliver 1 Gy to the rectal OAR, for pelvis small and medium imaging protocols. By expressing the results in terms of *“scans per Gray”*, the data can be more easily used by clinicians to justify imaging schedules. This is not meant to be a definitive value of acceptable additional dose; rather to give a meaningful method for making judgements in the context of individual patient plans and evaluation of concomitant doses.

**Table 4.** Summarising the maximum dose to the rectum for each imaging protocol and the resulting number of CBCTs that would be needed to deliver 1 Gy to the rectum [*Phantom dose measurements for Pelvis Medium are overestimated due to the size of the ATOM^®^ phantom representing a small patient.]

CBCT mode	Dose/no. of #	Max rectal dose (Gy) per CBCT scan	No. CBCT scans to deliver 1 Gy to OAR
**Pelvis S**	78 Gy/37#	0.007	143
**Pelvis M**	78 Gy/37#	0.02	50*

CBCT, cone beam CT; OAR, organ at risk.

## Conclusion

The aim of this work was to set out a simple method for implementing and optimising pelvis CBCT imaging protocols, based on patient attenuation, for the reduction in concomitant imaging doses. This work also addresses the importance of performing departmental dose (OAR and CTDIw100) measurements for each imaging CBCT mode and discusses how we can translate these results into more meaningful values for making patient group and per patient judgements and justification of additional imaging for individual patient plans. Our results show that it is entirely feasible for centres to optimise their current CBCT settings to vastly reduce the patient imaging doses. Justification of additional imaging is far easier for a clinician if the doses are lower and the risks to the patient fewer. By translating this risk to the Oncologist in terms of “No. scans per Gray” to achieve 1 Gy OAR tolerance, this allows for rapid, informed decisions regarding the imaging schedule and justification of concomitant doses.

The key points from this work are:

- New CBCT imaging modes for pelvis have been established for four patient groups (S/M/L/XL) based on planning scan CTDIvol dose indicator, given in the summary dose report;
- New imaging modes use a larger field of view and a full body contour image;
- Significant dose reductions of up to 83% for all modes except Pelvis XL, keeping doses ALARP;
- TrueBeam equivalent doses are approximately 50% less than Clinac iX doses.

For other centres still using the standard OBI imaging protocols, we hope that this work shows that it is a relatively simple method for optimising imaging protocols based on patient attenuation, requiring only equipment that is common to a radiotherapy department. The patient benefits in terms of dose reduction are vast in addition to the confidence gained within a department from having an in-depth understanding of imaging doses, especially given their ever increasing frequency in the future of radiotherapy.

## References

[1] Ariyaratne H, Chesham H, Alonzi R. Image-Guided radiotherapy for prostate cancer in the United Kingdom: a national survey. Br J Radiol 2017; 90: 20160059. doi: 10.1259/bjr.20160059 27925472 PMC5685109

[2] Ding GX, Alaei P, Curran B, Flynn R, Gossman M, Mackie TR, et al. Image guidance doses delivered during radiotherapy: quantification, management, and reduction: report of the AAPM therapy physics Committee task group 180. Med Phys 2018; 45: e84–99. doi: 10.1002/mp.12824 29468678

[3] Shah A, Aird E, Shekhdar J. Contribution to normal tissue dose from concomitant radiation for two common kV-CBCT systems and one MVCT system used in radiotherapy. Radiother Oncol 2012; 105: 139–44. doi: 10.1016/j.radonc.2012.04.017 22656240

[4] Alaei P, Spezi E, Reynolds M. Dose calculation and treatment plan optimization including imaging dose from kilovoltage cone beam computed tomography. Acta Oncol 2014; 53: 839–44. doi: 10.3109/0284186X.2013.875626 24438661

[5] Spezi E, Downes P, Jarvis R, Radu E, Staffurth J. Patient-Specific three-dimensional concomitant dose from cone beam computed tomography exposure in image-guided radiotherapy. Int J Radiat Oncol Biol Phys 2012; 83: 419–26. doi: 10.1016/j.ijrobp.2011.06.1972 22027261

[6] Abuhaimed A, Martin CJ, Sankaralingam M, Gentle DJ. A Monte Carlo investigation of cumulative dose measurements for cone beam computed tomography (CBCT) dosimetry. Phys Med Biol 2015; 60: 1519–42. doi: 10.1088/0031-9155/60/4/1519 25615012

[7] Abuhaimed A, Martin CJ, Sankaralingam M. A Monte Carlo study of organ and effective doses of cone beam computed tomography (CBCT) scans in radiotherapy. J Radiol Prot 2018; 38: 61–80. doi: 10.1088/1361-6498/aa8f61 28952463

[8] Alaei P, Spezi E. Imaging dose from cone beam computed tomography in radiation therapy. Phys Med 2015; 31: 647–58. doi: 10.1016/j.ejmp.2015.06.003 26148865

[9] Islam MK, Purdie TG, Norrlinger BD, Alasti H, Moseley DJ, Sharpe MB, et al. Patient dose from kilovoltage cone beam computed tomography imaging in radiation therapy. Med Phys 2006; 33: 1573–82. doi: 10.1118/1.2198169 16872065

[10] Ding GX, Coffey CW. Radiation dose from kilovoltage cone beam computed tomography in an image-guided radiotherapy procedure. Int J Radiat Oncol Biol Phys 2009; 73: 610–7. doi: 10.1016/j.ijrobp.2008.10.006 19147025

[11] Wood TJ, Moore CS, Saunderson JR, Beavis AW. Validation of a technique for estimating organ doses for kilovoltage cone-beam CT of the prostate using the PCXMC 2.0 patient dose calculator. J Radiol Prot 2015; 35: 153–63. doi: 10.1088/0952-4746/35/1/153 25634880

[12] Sawyer LJ, Whittle SA, Matthews ES, Starritt HC, Jupp TP. Estimation of organ and effective doses resulting from cone beam CT imaging for radiotherapy treatment planning. Br J Radiol 2009; 82: 577–84. doi: 10.1259/bjr/62467578 19255115

[13] Ding GX, Duggan DM, Coffey CW. Accurate patient dosimetry of kilovoltage cone-beam CT in radiation therapy. Med Phys 2008; 35: 1135–44. doi: 10.1118/1.2839096 18404948

[14] Sykes JR, Lindsay R, Iball G, Thwaites DI. Dosimetry of CBCT: methods, doses and clinical consequences. J. Phys.: Conf. Ser. 2013; 444: 012017. doi: 10.1088/1742-6596/444/1/012017

[15] Hess CB, Thompson HM, Benedict SH, Seibert JA, Wong K, Vaughan AT, et al. Exposure risks among children undergoing radiation therapy: considerations in the era of image guided radiation therapy. Int J Radiat Oncol Biol Phys 2016; 94: 978–92. doi: 10.1016/j.ijrobp.2015.12.372 27026304

[16] Wood TJ, Moore CS, Horsfield CJ, Saunderson JR, Beavis AW. *Accounting for patient size in the optimization of dose and image quality of pelvis cone beam CT protocols on the Varian OBI system*, *The British journal of radiology*. 2015; 88: 20150364. 10.1259/bjr.20150364 PMC4743457 26419892

[17] Langmack KA, Newton LA, Jordan S, Smith R. Cone beam CT dose reduction in prostate radiotherapy using Likert scale methods. Br J Radiol 2016; 89: 20150460. doi: 10.1259/bjr.20150460 26689092 PMC4986481

[18] IPEM Guidance on the establishment and use of diagnostic reference levels for medical X-ray examinations. Institute of Physics and Engineering in Medicine 2004;.

[19] R. I. G. R National, Image Guided Radiotherapy (*IGRT): Guidance for implementation and use*. 2012;.

[20] Alaei P, Spezi E. Commissioning kilovoltage cone-beam CT beams in a radiation therapy treatment planning system. J Appl Clin Med Phys 2012; 13: 19–33. doi: 10.1120/jacmp.v13i6.3971 PMC5718524 23149789

[21] Roxby P, Kron T, Foroudi F, Haworth A, Fox C, Mullen A, et al. Simple methods to reduce patient dose in a Varian cone beam CT system for delivery verification in pelvic radiotherapy. Br J Radiol 2009; 82: 855–9. doi: 10.1259/bjr/37579222 19289401

[22] Varian TrueBeam Technical Reference Guide - Volume 2: Imaging. 2019;.

[23] Hughes A. *Optimisation of Head and Neck Cone Beam CT for Image Guided Radiotherapy* MSc Thesis, Kings College. University of London 2017;.

[24] Neda Shiravand AH, Lavender F, MacDougall N. A simple method for measuring CBCT deterministic dose safety limits in radiotherapy., , Radiotherapy Physics, (2019;UKIO 2019Barts Health NHS Trust.

